# Laser-Assisted Drug Delivery for Hypertrophic Scar Treatment: A Scoping Review

**DOI:** 10.1093/jbcr/iraf167

**Published:** 2025-09-13

**Authors:** Maria Shilova, Karin Plummer, Robert Ware, Roy Kimble, Justin Clark, Esther Cho, Lucinda McMillan, Laura Kimble, Brandon Meikle, Lauren Kunde, Bronwyn Griffin

**Affiliations:** School of Nursing and Midwifery, Griffith University, Gold Coast 4111, Australia; School of Medicine and Dentistry, Griffith University, Gold Coast 4215, Australia; Queensland Children’s Hospital, South Brisbane 4101, Australia; School of Nursing and Midwifery, Griffith University, Gold Coast 4111, Australia; School of Medicine and Dentistry, Griffith University, Gold Coast 4215, Australia; School of Medicine and Dentistry, Griffith University, Gold Coast 4215, Australia; Queensland Children’s Hospital, South Brisbane 4101, Australia; Centre for Children’s Burns and Trauma Research, Queensland Children’s Hospital, South Brisbane 4101, Australia; Institute for Evidence-Based Healthcare, Bond University, Robina 4226, Australia; School of Medicine and Dentistry, Griffith University, Gold Coast 4215, Australia; School of Health and Rehabilitation Sciences, University of Queensland, St Lucia 4067, Australia; School of Biomedical Sciences, Faculty of Medicine, University of Queensland, St Lucia 4072, Australia; School of Biomedical Sciences, Faculty of Medicine, University of Queensland, St Lucia 4072, Australia; Centre for Children’s Burns and Trauma Research, Queensland Children’s Hospital, South Brisbane 4101, Australia; Children’s Health Research Centre, Faculty of Medicine, The University of Queensland, Herston 4006, Australia; Queensland Children’s Hospital, South Brisbane 4101, Australia; School of Nursing and Midwifery, Griffith University, Gold Coast 4111, Australia; Centre for Children’s Burns and Trauma Research, Queensland Children’s Hospital, South Brisbane 4101, Australia

**Keywords:** hypertrophic scar, keloid, laser-assisted drug delivery, fractional ablative laser, laser

## Abstract

Fractional ablative laser (FAL) is a minimally invasive method of hypertrophic scar management first introduced in 2004. Laser technologies and techniques have continued to evolve since that time and have included the addition of laser-assisted drug delivery (LADD) to augment the effects of the laser on scars. Laser-assisted drug delivery is increasingly reported in the literature and standard treatment protocols, underscoring the popularity of this technique among clinicians. Given this popularity, it is important to scrutinize evidence relating to the clinical outcomes LADD may achieve for patients. This scoping review examined literature relating to LADD for the treatment of hypertrophic scars in humans, aiming to clarify what clinical outcomes are achieved with its use and examining how these outcomes were studied and measured. *PubMed*, *EMBASE*, *Cochrane*, the *WHO International Clinical Trials Registry* and ClinicalTrials.gov were systematically searched, and data about study methodology, outcome measurement tools and results were extracted. Fifty-five publications that discussed LADD for the treatment of hypertrophic scars in humans were identified. Sixteen different substances, most frequently corticosteroids, were used for LADD treatment of hypertrophic scars, most often in conjunction with a carbon dioxide FAL. Study designs, outcome measurement strategies and follow-up time-frames were highly variable, as were the patient outcomes achieved. The clinical outcomes achieved with LADD are unclear, largely due to the variability of study methodology and outcome measurement. The efficacy of this technique requires further investigation with robustly designed, large trials which have comparison groups and use validated scar outcome measurement tools.

## INTRODUCTION

Hypertrophic scars can cause physical, developmental and psychosocial issues for patients. The structure of hypertrophic scars can cause contracture and tethering of surrounding tissue, which can be accompanied by chronic wounds and pruritis.^1^^,^^2^ These physical effects can then lead to a reduced ability to engage in activities of daily living, a poor quality of life, and delayed motor development in children.^1^^,^^3^^,^^4^ Furthermore, the presence of a scar can negatively impact self-perception, self-esteem, and hinder social interaction.^5–8^ In light of these wide-ranging impacts, there is a continuous pursuit for new and better treatments for people with hypertrophic scars.

**Figure 1:**
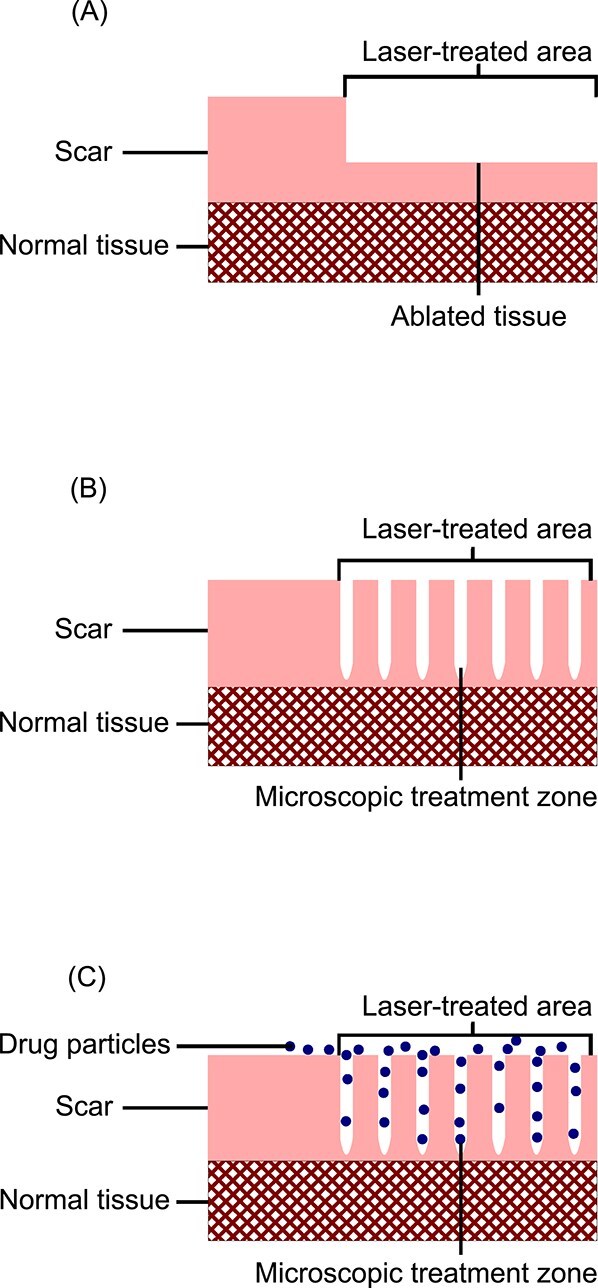
A Simplified Diagram of Ablative Lasers. (A) The result of scar treatment with an ablative laser. (B) The result of scar treatment with a FAL, which creates wells of ablated tissue (microscopic treatment zones) rather than ablating the entire superficial layer of skin. (C) The penetration of a compound into the scar tissue via LADD after treatment of the scar with a FAL

Laser technology is a rapidly developing and versatile tool for scar treatment. Several types of lasers are available, with ablative lasers being one broad category. Ablative lasers vaporize the tissue they encounter and prompt surrounding tissue to remodel (see Figure 1A), but are associated with risks of infection, scarring, pigment changes and a long postoperative recovery time.^9^^,^^10^ Fractionalization of the ablative laser in 2004 reduced these issues.^9^ Fractional ablative lasers (FALs) ablate small wells of tissue within the treatment area, which are known in the literature as both microscopic treatment zones and microthermal treatment zones (see Figure 1B).^10–13^ This action prompts the remodeling of the scar, while reducing operative risks and recovery time compared to non-FALs.^9^ There is ongoing technological development and research aiming to improve FAL scar treatment, including the development of adjunct treatments.

Laser-assisted drug delivery (LADD) is one adjunct treatment used with FAL. This idea was first presented in 2010, when fluorescently labeled methyl 5-aminolevulinate was applied to a scar after lasering, and was found to penetrate into the tissue via the ablated channels created by the FAL (see Figure 1C).^14^ Laser-assisted drug delivery allows for concurrent treatment of a scar with laser and medication without requiring painful intralesional injections of drugs, as was traditionally done. This technique was quickly adopted by the clinical community and has even been incorporated into some standard unit laser protocols, underscoring its popularity.^15–17^ However, the evidence for the efficacy of this technique for treating hypertrophic scars is still in its nascency.

There are a few randomized controlled trials (RCTs) investigating the use of LADD for hypertrophic scar treatment, and the existing ones employ varying LADD methodologies, have diverse follow-up time-frames and outcome measures, complicating the comparison of study results.^18^ Furthermore, some LADD techniques have appeared only in case reports or cohort studies, but have not been investigated in large, rigorous trials. A comprehensive understanding of which substances are used for LADD and what clinical outcomes are achieved is necessary for advancing both clinical practice and research endeavors.

This scoping review aims to identify evidence for treating hypertrophic scars with LADD in humans, to define the range of methodologies and outcome measures used in these studies, and to summarize patient outcomes.

## METHODS

This scoping review is based on a registered protocol, which was developed in accordance with Joanna Briggs Institute guidelines and the Preferred Reporting Items for Systematic Reviews and Meta-Analyses (PRISMA) extension for Scoping Reviews.^19–21^ The protocol was registered in OpenScience.^21^ We used the Systematic Review Accelerator software, which automates and facilitates systematic literature review, for all stages of the work.^22–25^

### Study eligibility criteria

This review included publications describing LADD for the treatment of hypertrophic scars in humans. The term “hypertrophic scar” included keloids in this review, as these entities are thought to lie on the same spectrum of the pathological scarring processes, and the clinical distinction between them can be ambiguous.^26^ Laser-assisted drug delivery was defined as the application of any substance onto a scar after treatment with a FAL, with the intention of treating the scar. Studies that used antibiotic ointments, impregnated dressings or emollients as dressings were excluded, because these agents are typically applied as part of a post-FAL wound care regimen, rather than for the treatment of scars.^27^^,^^28^ There were no restrictions on date or language.

### Search strategy

**Figure 2:**
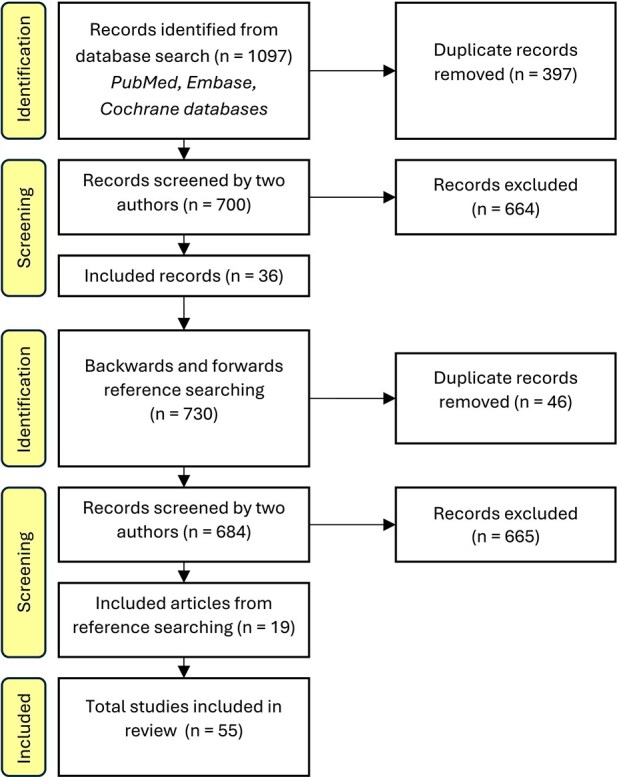
A Summarized PRISMA Flow Diagram for the Literature Search

The search strategy was designed by an experienced information specialist (see Supplementary File 1). The search was initially designed in PubMed using a combination of keywords and MeSH terms, then translated to other databases using the Polyglot Search Translator.^29^ The databases PubMed, Embase, and Cochrane CENTRAL were searched, as well as the WHO International Clinical Trials Registry and ClinicalTrials.gov, to maximize capture of unique relevant records.^30^ After removing duplicates, papers were screened separately by 2 authors, and disputes were resolved in consultation with a senior author (Figure 2 and Supplementary File 2). Forwards and backwards reference searching were performed on included articles.

### Data charting, quality appraisal, and data synthesis

A data charting tool was developed and piloted for agreement on 5 articles included in the primary literature search (see Table S1). Data charting was completed in a shared Microsoft Excel spreadsheet (Version 2312, Microsoft Corporation, Washington, United States) using the piloted tool.^31^ Attempts were made to obtain data missing from abstracts, but were unsuccessful.

The Mixed Methods Appraisal Tool (MMAT) was used to assess the quality of full-text publications containing data about the efficacy of LADD for hypertrophic scar treatment.^32^ The MMAT was designed to be used for literature reviews that include multiple study designs and can be used to assess 5 types of studies. Studies are assessed by 5 quality criteria specific to each study design, the fulfillment of the criteria being scored as “yes,” “no,” or “cannot tell.”^32^

The data were first examined as a single group and then in subgroups by the LADD compound used.

## RESULTS

### Publication characteristics

The search was completed in February 2023, yielding a total of 55 studies (see Figure 2): 37 original research papers, 5 case reports or small series, 2 technique papers, 1 clinical trial protocol, 1 book chapter and 9 abstracts (see Table 1).^13^^,^^33–86^ Forty-two studies reported outcomes related to the efficacy of LADD for treating hypertrophic scars. Laser-assisted drug delivery was most often described within the study methodology, rather than being the investigatory aim of the paper. One publication was an RCT protocol, hence, it did not have any results.^34^ The remaining 12 publications either contained a description of LADD as a scar treatment technique (eg, Benzaquen et al.’s technique paper)^86^ or had cohorts where not all participants received LADD and where the results of the LADD subgroup were not separately reported.^42^^,^^45–47^^,^^49^^,^^53^^,^^58^^,^^69^^,^^74^^,^^79^^,^^85^^,^^86^

**Table 1:** Summary of Literature Concerning Laser-Assisted Compound Delivery for the Treatment of Hypertrophic Scars

**First author, Year of publication, Country**	**Study type**	**Sample and group characteristics** Age in years F = female, M = male		**Interventions, dose and concentration of laser-assisted-drug-delivery compound used**	**LADD substance used**	**Total MMAT score (*x*/5)** ^a^	**Outcome measures**	**Relevant summarized outcomes**
Abd El-Dayem,^50^ 2020, Egypt	Nonrandomized, comparative, split-scar study	*n* = 30 Age $\overline{x}$ = 26.4 Sex = not stated		Intervention 1: Er:YAG FAL LADD Intervention 2: intralesional triamcinolone 4 treatments per participant, 4 weeks apart	Betamethasone	1	VSS	Improvement^b^ of VSS in both groups compared to baseline, no significant difference between groups
Al Janahi,^75^ 2019, USA	Case study	*n* = 1 Age = 72 M		CO_2_ FAL LADD, then further triamcinolone injected intralesionally	Triamcinolone	3	VSS	VSS reduced after treatment
Almukhtar,^62^ 2018, USA	Case series (abstract)	*n* = 4 Age range 3-44 Sex not stated		CO_2_ FAL LADD (40 mg/mL, dose not stated) + pulse dye laser 1-4 treatments per participant, 3-12 months apart	Triamcinolone	N/A	Not stated	Improvement in scar appearance, erythema, texture and symptoms in all participants
Bazargan,^34^ 2023, Iran	Protocol for a randomized controlled trial (abstract)	Recruitment commenced Jan 2023		Intervention: CO_2_ FAL LADD Control: pulsed dye laser 3 treatments per participant	5-fluorouracil	N/A	VSS	N/A
Behrangi,^55^ 2018, Iran	Nonrandomized, prospective within-patient controlled study (within-participant comparison on 2 separate scars)	*n* = 33 Age $\overline{x}$ = 28.8 63.6% F, 36.4% M		Intervention: CO_2_ FAL LADD Control: intralesional triamcinolone 2-8 treatments per participant	Triamcinolone	1	Examination of scar photographs^c^	Improvement^b^ in general scar appearance and hypertrophy in intralesional triamcinolone group compared to LADD group. Improvement^b^ in dyschromia and texture in LADD group compared to intralesional triamcinolone group.
Benzaquen,^86^ 2019, France	Technique paper	N/A		Describes the use of triamcinolone CO_2_ FAL LADD and intralesional triamcinolone during the same procedure	Triamcinolone	N/A	N/A	
Blome-Eberwein,^77^ 2018, USA	Randomized controlled trial (within-participant comparison on 2 separate scars) (abstract)	*n* = 28 Age $\overline{x}$ = 39.4 46.4% F, 53.6% M		Intervention: CO_2_ FAL LADD Control: CO_2_ FAL 5 treatments per participant	Unspecified steroid	N/A	VSS, POSAS, ultrasound, sensation, elasticity, pigment and erythema evaluation	Improvement in all measures for both groups, except erythema. LADD improved elasticity and pigmentation significantly more than laser alone (no statistical details included).
Burns,^37^ 2017, USA	Case report	*n* = 1 Age = 50s M		CO_2_ FAL LADD Single treatment	Triamcinolone	3	Nil	Subjective improvement in pruritis, scar pliability and range of motion
Cavalié,^43^ 2014, France	Retrospective cohort study	*n* = 23 Age $\overline{x}$ = 31.4 39.1% F, 60.9% M		Er:YAG FAL LADD 3-29 treatments per participant	Betamethasone	3	Examination of photographs^c^, patient satisfaction via visual analog scale^c^	Improvement in all participants, satisfaction rate median 7 (range 3-10), recurrence in 22% participants at mean 8 months posttreatment
Dai,^52^ 2021, China	Retrospective cohort study	*n* = 50	*n* = 31 Age $\overline{x}$ = 41 38.7% F, 61.3% M	Intervention 1: CO_2_ FAL LADD 7 treatments per participant, 1 month apart	Platelet-rich plasma	2	VSS, University of North Carolina 4P Scar Scale	Improvement^b^ in VSS scores and UNC4P scores in both groups. Reductions in VSS and UNC4P scores at 3 and 6 month follow-up are greater^b^ in the LADD group than the FAL group.
			*n* = 19 Age $\overline{x}$ = 40 57.9% F, 43.1% M	Intervention 2: CO_2_ FAL 7 treatments per participant, 1 month apart	–			
Deng,^72^ 2020, China	Randomized comparative trial (abstract)	*n* = 30 Age not stated Sex not stated		Intervention 1: Er:YAG FAL LADD Intervention 2: Er:YAG FAL LADD + broad-band light Intervention 3: CO_2_ FAL LADD	Asaticoside	N/A	VSS	Reduction^b^ of VSS in all groups, more in groups without broad-band light. All groups had 100% “mitigation rates” of pain and pruritis
Elrod,^54^ 2020, Switzerland	Retrospective cohort study	*n* = 17 Age $\overline{x}$ = 11.37 53% F, 47% M		CO_2_ FAL LADD ± pulsed dye laser Up to 6 treatments per participant, minimum 6 weeks apart	Triamcinolone	4	VSS, POSAS, Itch Man Scale	Improvement^b^ of VSS and POSAS scores in parameters at the end of the treatments. Improvement^b^ of Itch Man Scale scores at the end of the treatments.
Ge,^33^ 2021, China	Retrospective cohort study	*n* = 21 Age $\overline{x}$ = 31.4 61.9% F, 38.1% M		CO_2_ FAL LADD Average number of treatments 4.86 ± 1.74, 12 weeks apart	MEBO	2	The study included scars ≥20% TBSA, not all of which were hypertrophic. Results for hypertrophic scars were not reported separately.	
Greywal,^85^ 2017 USA	Technique paper	Not applicable		Describes a technique to treat hypertrophic earlobe scars, which includes CO_2_ FAL LADD	Triamcinolone	N/A	N/A	
Han,^58^ 2022, South Korea	Randomized controlled trial	*n* = 9 Age $\overline{x}$ = 31.56 44.4% F, 55.56% M		Intervention 1: Erbium:yttrium-scandium-garnet-gallium FAL LADD Single treatment	DA-5520 LADD (contains allantoin, heparin sodium and dexpanthenol)	3	No relevant outcomes about the efficacy of LADD for treating hypertrophic scars: the DA-5520 LADD group did not have participants with hypertrophic acne scars.	
		*n* = 9 Age $\overline{x}$ = 26.44 66.67% F, 33.33% M		Control 1: Erbium:yttrium-scandium-garnet-gallium FAL alone Single treatment				
		*n* = 9 Age $\overline{x}$ = 27.11 11.11% F, 88.89% M		Intervention 2: comedone extraction and topical DA-5520				
		*n* = 9 Age $\overline{x}$ = 26.89 33.33% F, 66.67% M		Control 2: comedone extraction alone				
Har-Shai,^53^ 2020, Germany	Case report	*n* = 1 Age = 50 F		Resistant hypertrophic facial scars treated with cryotherapy + pulsed dye laser + CO_2_ FAL LADD	Triamcinolone + 5-FU + Hyaluronidase	N/A	No relevant outcomes about the efficacy of LADD for treating hypertrophic scars: this work described a technique used for a difficult case, with no outcome measures completed.	
Issler-Fisher,^45^ 2017, Australia	Prospective cohort study	*n* = 47 Age $\overline{x}$ = 34 72% F, 28% M		CO_2_ FAL ± LADD or intralesional triamcinolone ± surgical reconstructive procedures Single treatment	Triamcinolone	N/A	Not all participants received LADD, and no subgroup data were reported for participants who received LADD	
Issler-Fisher,^49^ 2020, Australia	Retrospective cohort study	*n* = 412 Age $\overline{x}$ = 37 58.7% F, 41.3% M		CO_2_ FAL ± LADD or intralesional triamcinolone 1-4 treatments per participant	Triamcinolone	N/A	Not all participants received LADD, and no subgroup data were reported for participants who received LADD	
Issler-Fisher,^69^ 2020, Australia	Retrospective cohort study	*n* = 78 Age $\overline{x}$ = 40 59% F, 41% M		CO_2_ FAL ± LADD or intralesional triamcinolone Single treatment	Triamcinolone	N/A	Not all participants received LADD, and no subgroup data were reported for participants who received LADD	
Issler-Fisher,^47^ 2021, Australia	Retrospective nested case control study	*n* = 187 Age $\overline{x}$ = 39 59.9% F, 40.1% M		CO_2_ FAL ± LADD or intralesional triamcinolone ≥1 treatment per participant	Triamcinolone	N/A	Not all participants received LADD, and no subgroup data were reported for participants who received LADD	
Kauvar,^63^ 2011, Belgium	Prospective cohort study (abstract)	*n* > 100 Age not stated Sex not stated		Intervention: 1440 nm or 1540 nm fractional (unclear if ablative or nonablative) laser LADD 1-4 treatments per participant, 4-6 weeks apart	Unspecified costricosteroid	N/A	No relevant outcomes about efficacy of LADD for treating hypertrophic scars: interim results only reported in the abstract	
Khandelwal,^67^ 2014, USA	Prospective cohort study	*n* = 44 Age $\overline{x}$ = 18 45.5% F, 54.5% M		CO_2_ FAL LADD 1-4 treatments per participant	Triamcinolone	1	VSS	VSS was recorded in 90% participants. Improvement^b^ of VSS scores at the end of treatments.
Krakowski,^44^ 2014, USA	Case series	*n* = 2 Age = 2, 18 50% F, 50% M		CO_2_ FAL LADD (1 participant only) 1 treatment	Triamcinolone	2	Range of motion of affected digits	Improved range of motion in both participants
Lei,^73^ 2017, China	Prospective cohort study	*n* = 158 Age $\overline{x}$ = 30.32 62% F, 38% M		CO_2_ FAL with two-pass manual fraction technology LADD Average 2.92 treatments per participant	MEBO	0	VSS, University of North Carolina (UNC) Scar Scale, intraoperative pain score (0-9, 9 indicating severe pain)^c^, Patient satisfaction score (1-4, 4 indicating very satisfied)^c^	Improvement^b^ of VSS and UNC scores at the end of treatments
Lin,^48^ 2021, Taiwan	Randomized controlled split-scar study	*n* = 12 Age $\overline{x}$ = 37.2 100% F, 0% M		Intervention: CO_2_ FAL LADD Control: CO_2_ FAL 5 treatments per participant, 4 weeks apart	Clobetasol propionate	3	POSAS, examination of photographs by doctors and scoring the difference between sides on a numerical rating scale 0-10 (0 being “totally identical,” 10 being “significantly different”). Doctors also scored the scar appearance on photos with a numerical rating scale 1-10 (1 being “normal skin,” 10 being “worst appearance”)^c^	No statistically significant improvement of POSAS scores in either group 7 months after the last treatment, and no statistically significant difference in POSAS scores between the treatment and control.
Lin,^61^ 2022, Taiwan	Prospective cohort study	*n* = 10 Age $\overline{x}$ = 37.7 100% F		CO_2_ FAL LADD 5 treatments per participant, 4 weeks apart	Clobetasol propionate	3	POSAS, examination of photographs by doctors and scoring appearance on a numerical rating scale 1-10 (1 being “normal skin,” 10 being “worst appearance”)^c^	Improvement^b^ in all parameters of POSAS scores, and on examination of photographs by 3 doctors.
Liu,^65^ 2020, China	Randomized controlled trial	*n* = 30 Age $\overline{x}$ = 38.7 46.6% F, 53.3% M		Intervention: CO_2_ FAL LADD + pulsed dye laser 2 treatments of CO_2_ FAL LADD, 3 months apart 6 treatments of pulsed dye laser, 1 month apart	MEBO (containing sesame oil, b-sitosterol, berberine, and other Chinese herbal plant ingredients)	0	VSS, pain (visual analog scale), pigmentation scores^c^	Improvement^b^ in VSS in both groups, but significantly more in the LADD group, compared to the control group. Less^b^ postprocedural pain in the LADD group, compared to the control group
		*n* = 31 Age $\overline{x}$ = 37.9 48.4% F, 51.6% M		Control: CO_2_ FAL + pulsed dye laser 2 treatments of CO_2_ FAL, 3 months apart 6 treatments of pulsed dye laser, 1 month apart				
Liu,^82^ 2022, China	Retrospective cohort (nested case–control) study	*n* = 42 Age $\overline{x}$ = not stated; range 7-28 y/o 55% F, 45% M		Intervention 1: CO_2_ FAL LADD Average of 2.65 treatments per participant, 4-6 weeks apart	Betamethasone	2	Burns Specific Health Scale-Brief (BSHS-B), 36-Item Short Form Health Survey (SF-36), Pittsburg Sleep Quality Index (PSQI), POSAS, University of Carolina 4P Scars Scale (UNC4P), Douleur Neuropathique 4 questions (DN4)	The study’s aim was to compare quality-of-life outcomes in those undergoing CO_2_ FAL treatment of hypertrophic scars versus those undergoing scar reconstruction. Better^b^ BSHS-B, SF-36 and PSQI scores in the CO_2_ FAL group than the conventional surgery group. Better^b^ POSAS total scores, DN4 and UNC4P scores in the CO_2_ FAL group than the comparator group, at the end of the treatment period (no pretreatment comparative measures taken).
		*n* = 23 Age $\overline{x}$ = 42.30 43% F, 57% M		Intervention 2: surgical scar reconstruction Average of 2.61 procedures per participant				
Lueangarun,^41^ 2020, Thailand	Randomized controlled split-scar study (abstract)	A total of 15 participants with 24 scars, group characteristics not stated		Intervention: CO_2_ FAL LADD Control 1: liquid silicone gel Control 2: CO_2_ FAL	Liquid silicone gel	N/A	POSAS, VSS, Standardized photography biometric measurements (no further detail)	More improvement^b^ in VSS and POSAS scores in CO_2_ FAL LADD group compared to topical liquid silicone gel or FAL alone.
Lv,^13^ 2020, China	Nonrandomized comparative trial	*n* = 68 Age not stated 37% F, 63% M	*n* = 35 Age $\overline{x}$ = 38.60 23% F, 77% M	Intervention: CO_2_ FAL LADD Average of 2.43 treatments per participant, 4-12 weeks apart	Betamethasone	2	Pittsburg Sleep Quality Index (PSQI), Visual Analog Scale (pain, pruritis), Brief Pain Inventory, 5-D Itch Scale, Four-item itch questionnaire, Objective sleep parameters: electrocardiogram, cardiopulmonary coupling software	Lower PSQI scores in CO_2_ FAL LADD group after treatments were completed, indicating better sleep quality. Lower pain scores (both measures) and pruritis scores (all measures) in CO_2_ FAL LADD group after treatments completed.
			*n* = 33 Age $\overline{x}$ = 43.06 52% F, 48% M	Comparison: Conventional surgical treatment of scars (eg, excision)				
Majid,^80^ 2017, India	Prospective cohort study	*n* = 10 Age $\overline{x}$ = 9.7 70% F, 30% M		CO_2_ FAL LADD 3-5 treatments per participant, 1 month apart	Triamcinolone	1	VSS, Physician Global Assessment score^c^	Improvement^b^ in total VSS and sub-scale scores (except for vascularity) compared to pretreatment measurements. 6/10 patients had an excellent response by the Physician Global Assessment score, 3/10 had a good response, and one had a poor response.
Maninder,^35^ 2022, India	Retrospective cohort study	*n* = 42 Age $\overline{x}$ = not stated, range 7-28 years old 70% F, 30% M		CO_2_ FAL LADD 1-3 treatments per participant	Fluticasone propionate	1	Investigator Global Assessment (IGA)^c^, Patient Global Assessment (PGA)^c^	The study included both atrophic and hypertrophic scars. Results for hypertrophic scars (*n* = 29) only: Mean 30.86% improvement in IGA, and 44.31% improvement in PGA scores.
Manuskiatti,^51^ 2017, Thailand	Randomized comparison split-scar study (abstract)	*n* = 20 Age not stated Sex not stated		Intervention 1: Er:YAG FAL LADD Intervention 2: Er:YAG FAL 4 treatments per participant, 2 weeks apart	Clobetasol	N/A	POSAS, scar thickness (unknown method)	No statistically significant difference in outcomes between comparison groups.
Manuskiatti,^57^ 2021, Thailand	Randomized controlled split-scar trial	*n* = 19 Age $\overline{x}$ = 34 100% F		Intervention: Er:YAG FAL LADD Control: Er:YAG FAL and topical petrolatum 4 treatments per participant, 2 weeks apart	Clobetasol	3	POSAS, scar height (calipers)^c^	No statistically significant difference in outcomes between comparison groups.
Miletta,^70^ 2019, USA	Case series (abstract)	Not stated		CO_2_ FAL LADD	Polylactic-co-glycolic acid impregnated with triamcinolone crystals	N/A	Examination of scar photographs^c^	Improvement in hypertrophy at 8-week follow-up (no further details provided)
Ouyang,^84^ 2018, China	Randomized controlled trial	*n* = 56 Age range 3- 51 37.5% F, 62.5% M	*n* = 28 Age and sex not stated	Intervention 1: pulse dye laser, CO_2_ fractional laser with LADD 2 treatments per participant, 3 months apart	MEBO	1	VSS	The aim of the study was to evaluate the efficacy of PDL vs PDL with FAL. Relevant outcomes: More improvement^b^ in VSS score in FAL group compared to pulsed dye laser group.
			*n* = 28 Age and sex not stated	Intervention 2: pulse dye laser with topical MEBO afterwards 2 treatments per participant, 1 month apart				
Park,^36^ 2017, South Korea	Nonrandomized prospective split-scar comparative study	*n* = 10 age range 31-42 90% F, 10% M		Intervention 1: Er:YAG FAL LADD Intervention 2: Er:YAG FAL and intralesional triamcinolone	Desoxymethasone	2	VSS, intraprocedural pain (visual analog scale), pruritis (visual analog scale), patient satisfaction using a 4-point scale^c^	No statistically significant difference in scar outcomes between groups. Less intra-procedural pain in Er:YAG FAL LADD group than comparator group.
Patel,^74^ 2019, USA	Prospective cohort study	*n* = 49 Age $\overline{x}$ = 4.86 46.9% F, 53.1% M		CO_2_ FAL LADD to most scars (exact number not stated) after CO_2_ FAL treatment	Triamcinolone	N/A	POSAS	The aim of the study was “to further elucidate the clinical role of CO_2_ FAL therapy for pediatric hypertrophic burn scars.” Outcomes were not separately reported for subgroup that had LADD in addition to CO_2_ FAL.
Poetschke,^46^ 2020, Unknown	Book chapter	*n* = 1 Age = 23 M		A teaching scar case study is presented, which involves LADD	5-FU + Unspecified steroid	N/A	No relevant outcomes	
Sabry,^56^ 2019, Egypt	Randomized comparative trial	*n* = 10 Age $\overline{x}$ = 22.3 60% F, 40% M		Intervention 1: CO_2_ FAL LADD	Verapamil	2	POSAS (pain, pruritis and overall satisfaction ratings only), VSS	More improvement^b^ in VSS vascularity sub-score, total VSS score and POSAS overall satisfaction score in both LADD groups compared to FAL alone, but no difference between LADD groups. More improvement^b^ in VSS height and pliability sub-scores in 5-FU LADD group, compared to FAL alone.
		*n* = 10 Age $\overline{x}$ = 12.6 40% F, 60% M		Intervention 2: CO_2_ FAL LADD	5-FU			
		*n* = 10 Age $\overline{x}$ = 24.35 50% F, 50% M		Intervention 3: CO_2_ FAL All groups: 4 treatments per participant, 1 month apart	N/A			
Sabry,^60^ 2020, Egypt	Randomized (split-scar) comparative trial	*n* = 20 Age $\overline{x}$ = 12.5 15% F, 85% M		Intervention 1: CO_2_ FAL LADD Intervention 2: intralesional BTXA 1 treatment per participant	BTXA	1	POSAS (patient scar assessment scale only), VSS	Outcomes in the study were reported separately for “keloid” and “hypertrophic” scars. Hypertrophic scars: More improvement^b^ in total VSS score in LADD group than the comparator group, but no statistically significant superiority if VSS subscores are compared. Keloid scars: More improvement^b^ in vascularity and pliability in LADD group compared to the comparator group. For both hypertrophic scars and keloids: Better^b^ POSAS patient scar assessment scale score in CO_2_ FAL LADD group than comparator group.
Saraiva,^39^ 2018, Brazil	Prospective cohort study	*n* = 18 Age $\overline{x}$ = 38.93 100% F, 0% M		Er:YAG FAL LADD 4 treatments per participant, 15 days apart	Triamcinolone	1	Examination of photographs and assignment of percentage global improvement^c^	35.53% of patients showed 0%-25% improvement, 37.80% showed 25%-50% improvement, 8.89% showed 50%-75% improvement, and 17.80% showed 75%-100% improvement.
Sullivan,^59^ 2021, Australia	Case report (abstract)	*n* = 1 Unknown		(Unknown FAL) LADD	5-FU + Triamcinolone	N/A	Not stated	No details about specific case outcomes. Conclusion of abstract is that triamcinolone and 5-fluorouracil “LADD shows both efficacy and esthetic benefits.”
Tan,^76^ 2020, China	Retrospective cohort study	*n* = 221 Age $\overline{x}$ = 33.6 46.6% F, 53.4% M		CO_2_ FAL LADD ≥1 treatment per participant, 1 month apart	Triamcinolone	2	VSS, durometry, spectrocolorimetry (redness and lightness)	The aim of the study was to investigate the efficacy and safety of CO_2_ FAL for hypertrophic scars and compare early versus late intervention. Participants were divided into subgroups by hypertrophic scar age (since original injury). Relevant outcomes: Improved^b^ of VSS scores, compared to pretreatment. Improvement^b^ in scar hardness, compared to pretreatment in all subgroups (except scars 6-12 months old). Improvement^b^ in scar lightness, compared to pretreatment in all subgroups (except scars <1 month old and 3-6 months old). Improvement^b^ in scar redness, compared to pretreatment, in all subgroups (except scars 3-6 months old).
Tawfik,^78^ 2019, Egypt	Randomized comparative split-scar trial	*n* = 24 Age $\overline{x}$ = 26 41.7% F, 58.3% M		Intervention 1: Er:YAG FAL LADD 1 treatment per participant Intervention 2: topical 5-FU cream	5-FU	2	VSS, scar size (length, height, width, measured with calipers)^c^	VSS results statistically inconclusive. Greater reduction^b^ of scar height in LADD group, compared to topical 5-FU group.
Tu,^38^ 2018, USA	Case series	*n* = 2 Age = 5, 11 100% F, 0% M		CO_2_ FAL LADD	Triamcinolone	1	Range of motion of affected digits	The aim of the study was to describe the use of CO_2_ FAL for hypertrophic scars caused by treadmill friction injuries. Relevant outcomes: One participant had improved range of motion after laser treatment (R correlation >0.9), and the other participant had subjective improvement.
Waibel,^83^ 2013, USA	Prospective cohort study	*n* = 15 Age $\overline{x}$ = 33.6 80% F, 20% M		CO_2_ LADD ± pulse dye laser Some are combined with shave excision or pulsed dye laser procedures 3-5 treatments per participant, 2-3 months apart	Triamcinolone	2	Examination of photographs and rating on 4-point scale for parameters of dyschromia, hypertrophy, texture and overall appearance	Overall appearance scores improved by an average 2.73 (out of 3 points), texture improved by an average 2.84 points, hypertrophy improved by an average of 2.76, and dyschromia improved by an average of 2.36 points. No statistical significance reported.
Waibel,^64^ 2013, USA	Prospective comparative study (abstract)	A total of 20 participants, but unclear if this is a split-scar or 2-group study. Further group characteristics are not stated		Intervention 1: (unspecified FAL) LADD Intervention 2: (unspecified FAL) LADD 3 treatments per participant, 1 month apart in both groups	Triamcinolone 5-FU	N/A	Caliper measurement of scar size, examination of scar photographs^c^	3 months posttreatment: average decrease in scar height by 0.415 mm, and average length decrease by 0.455 mm, no statistical difference between groups. Width of the scar increased in the triamcinolone LADD group.
Waibel,^81^ 2019, USA	Nonrandomized comparative trial (within-patient comparison either via 2 different scars or split-scar)	*n* = 20 Age not stated Sex not stated		Intervention 1: CO_2_ FAL LADD Intervention 2: CO_2_ FAL LADD 3 treatments per participant, 1 month apart in both groups	Triamcinolone 5-FU	1	Caliper measurement of height, length, width^c^	No statistically significant difference in outcomes between groups.
Wang,^66^ 2020, China	Prospective cohort study	*n* = 41 Age $\overline{x}$ = 27.4 51% F, 49% M		CO^2^ FAL LADD 8 treatments per participant, 4 weeks apart	Triamcinolone	4	POSAS	Improvement^b^ in all POSAS parameters at 1 month posttreatment and 24 months posttreatments, compared to pretreatment.
Yan,^40^ 2020, China	Randomized comparative trial	*n* = 16 Age $\overline{x}$ = 23.25 12.5% F, 87.5% M		Intervention 1: Microneedling and topical 5-aminolevulenic acid	N/A	1	VSS	No statistically significant difference in outcomes between groups.
		*n* = 28 age $\overline{x}$ = 25.29 14.3% F, 85.7% M		Intervention 2: CO_2_ FAL LADD 3 treatments per participant, 1 month apart	5-amnolevulenic acid			
		*n* = 8 age $\overline{x}$ = 23.25 37.5% F, 62.5% M		Intervention 3: intralesional betamethasone	N/A			
Ządkowski,^71^ 2016, Poland	Prospective cohort study	*n* = 47 age $\overline{x}$ = 10.5 55% F, 45% M		CO_2_ FAL LADD 1-2 treatments per participant	Allantoin	0	VSS, scar thickness (ultrasound)	The aim of the study was to present the technique used for hypertrophic scar treatment used by the authors. Results inconclusive due to reporting.
Zhang,^68^ 2022, China	Randomized comparative trial	*n* = 40 Age $\overline{x}$ = not stated %F %M not stated		CO_2_ FAL LADD 4 participant groups, all treated with CO2 FAL LADD, groups differing by energy and density settings for the FAL.	Compound heparin sodium and allantoin gel	0	VSS	The aim of the study was to analyze and compare the effect of different energy and density parameters on the treatment of hypertrophic burn scars. Relevant outcomes for efficacy of LADD for treating hypertrophic scars: Improvement^b^ of VSS scores in all groups, compared to pretreatment.
Zuccaro,^79^ 2018, Canada	Retrospective cohort study	*n* = 125 Age $\overline{x}$ = 6.62 36.8% F, 63.2% M		CO_2_ LADD FAL and/or pulsed dye laser	Triamcinolone	N/A	VSS, Toronto Pediatric Itch Scale	Participants could receive either CO_2_ FAL LADD or pulsed dye laser, or both lasers. Outcomes were reported for the whole group only, and outcomes for the laser subgroups were not reported.
Zuccaro,^42^ 2021, Canada	Prospective cohort study	*n* = 20 Age $\overline{x}$ = 5.89 35% F, 65% M		CO_2_ LADD FAL and/or pulsed dye laser ≥3 treatments per participant, 2 months apart	Triamcinolone	N/A	VSS, POSAS (observer only), vascularity, elasticity, melanin pigmentation, scar thickness (ultrasound)	Participants could receive either CO_2_ FAL LADD or pulsed dye laser, or both lasers. Outcomes were reported for the whole group only, and outcomes for the laser subgroups were not reported.

^a^Quality assessment only completed for full-text articles investigating the efficacy of LADD in treating hypertrophic scars.

^b^Statistically significant.

^c^Unvalidated outcome measure.

Abbreviations: CO_2_, carbon dioxide, Er:YAG, erbium-doped yttrium-aluminum-garnet; FAL, fractional ablative laser; MMAT, mixed methods appraisal tool.

Corticosteroids were the most common LADD substances used (*n* = 37/55, 67%), although a total of 16 different substances (*n* = 18 if combinations of substances are counted separately) were used for LADD scar treatment (see Table 1). The literature for each LADD compound is described separately below.

### Study quality

No studies met all 5 MMAT quality criteria (see Table 1), and many had criteria that could not be assessed due to a lack of information (see Table S2).

### Outcome measurement tools

The efficacy of LADD for treating hypertrophic scars was assessed using various outcome measures (see Table 1). Validated scar assessment scales, such as the Patient and Observer Scar Assessment Scale (POSAS) and Vancouver Scar Scale (VSS) were used in 30 studies (71%).^34^^,^^36^^,^^40–42^^,^^48^^,^^50–52^^,^  ^54^^,^^56^^,^^57^^,^^60^^,^^61^^,^^65–68^^,^^71–80^^,^^82^^,^^84^ In 6 of these, the scales were not used as designed.^36^^,^^50^^,^^56^^,^^60^^,^^61^^,^^78^ For example, 2 studies used the VSS, but scar assessment was done on photographs, rather than with a clinical examination.^36^^,^^50^

Three studies used nonvalidated numerical rating scales to assess patient satisfaction with their laser treatment.^36^^,^^43^^,^^73^

Twelve studies used objective single-parameter measurement tools, such as goniometers for measuring range of motion, or ultrasound for scar thickness measurements.^13^^,^^38^^,^^42^^,^^44^^,^^51^^,^^57^^,^^64^^,^^71^^,^^76–78^^,^^81^ Not all of these were the gold standard for the scar parameter measured.

Other outcome measurement tools included subjective assessment of photographs by clinicians (*n* = 7/42 publications, 17%), unvalidated global assessment scales (*n* = 2/42 publications, 5%), and visual analog scales to assess intraprocedural pain and pruritis (*n* = 3/42 publications, 7%).^13^^,^^35^^,^^36^^,^^39^^,^^43^^,^^48^^,^^55^^,^^61^^,^^65^^,^^70^^,^^80^^,^^83^ One study also gave unvalidated numerical scores of “pigmentation rate,” with insufficient detail provided for the replication of this measurement.^65^ Two studies (an abstract of a case series and a case report) reported subjective improvement in scar characteristics without scales or objective instruments.^37^^,^^62^

### Corticosteroid LADD

Thirty-seven publications used corticosteroids to treat hypertrophic scars via LADD.^13^^,^^35–39^^,^^42–45^^,^^47–51^^,^^54^^,^^55^^,^^57^^,^^61–64^^,^^66^^,^^67^^,^  ^69^^,^^70^^,^^74–77^^,^^79–83^^,^^85^^,^^86^ The most common corticosteroid used was triamcinolone (*n* = 25/37, 68%), followed by clobetasol (*n* = 4/37, 11%), betamethasone (*n* = 4/37, 11%), desoxymethasone (*n* = 1/37, 3%) and fluticasone (*n* = 1/37, 3%). Most publications (*n* = 29/37, 78%) used a carbon dioxide (CO_2_) FAL to deliver the corticosteroid (see Table 1).

Ten studies compared the clinical efficacy of corticosteroid LADD for treating hypertrophic scars to another intervention (see Table 1).^13^^,^^36^^,^^50^^,^^51^^,^^55^^,^^57^^,^^64^^,^^77^^,^^81^^,^^82^ More than half of these studies found improved scar outcomes in both of their groups, but no statistically significant difference between them.^36^^,^^50^^,^^51^^,^^57^^,^^64^^,^^81^ The exceptions to this were Behrangi et al. and Blome-Eberwein et al., who found that LADD improved specific scar parameters significantly more than intralesional triamcinolone or laser alone (see Table 1).^55^^,^^77^ Liu et al. and Lv et al. also found that CO_2_ FAL LADD improved quality of life outcomes more than conventional surgery did.^13^^,^^82^ However, the comparability of their groups at baseline is unclear, as both only conducted quality of life and scar assessments posttreatment, and laser treatment was allocated to individuals with smaller or less severe scars. Lin et al., investigated if clobetasol ointment LADD could prevent the formation of thyroidectomy scars if used early postthyroidectomy, and compared this to FAL alone. They found that neither intervention prevented scarring, and no outcome differences between the groups.^48^

Sixteen single-group studies investigated corticosteroid LADD efficacy for treating hypertrophic scars.^35^^,^^37–39^^,^^43^^,^^44^^,^^54^^,^  ^61^^,^^62^^,^^66^^,^^67^^,^^70^^,^^75^^,^^76^^,^^80^^,^^83^ All stated that there were improvements in scar characteristics following their treatment protocol, however, there was variation in laser/LADD compound combination were used, how outcomes were measured and degree of improvement reported (see Table 1).

Ten publications did not report any outcomes. These included 2 technique papers, which described scar management strategies that included triamcinolone LADD, an abstract with incomplete results, and 7 studies where LADD was used for part of the cohort, but where subgroup analysis for these participants was not reported.^42^^,^^45^^,^^47^^,^^49^^,^^63^^,^^69^^,^^74^^,^^79^^,^  ^85^^,^^86^

### 5-Fluorouracil LADD

Three full-text publications, 1 abstract and 1 RCT protocol investigated the efficacy of 5-fluorouracil (5-FU) LADD scar treatment (see Table 1).^34^^,^^56^^,^^64^^,^^78^^,^^81^ Four studies used a CO_2_ FAL, and one used an erbium-doped yttrium-aluminum-garnet (Er:YAG) FAL.^34^^,^^56^^,^^64^^,^^78^^,^^81^

The 3 full-text publications compared 5-FU LADD to another scar treatment intervention. Waibel et al. found that 5-FU LADD and triamcinolone LADD both produced improvements in VSS scores, but without a statistically significant difference between them.^64^^,^^81^ Sabry et al. similarly found that while 5-FU LADD was better than laser alone, it only surpassed its comparator, verapamil LADD, in reduction of height and pliability VSS subscores.^56^ Tawfik et al. found that 5-FU LADD reduced scar height significantly more than topical 5-FU, but used calipers to measure this.^78^ Their VSS results are inconclusive.^78^

### Moist exposure burn ointment LADD

Four publications described the use of moist exposure burn ointment (MEBO) as a LADD agent with a CO_2_ FAL (see Table 1).^33^^,^^65^^,^^73^^,^^84^ Of these, 2 studies compared the efficacy of MEBO LADD to another intervention: one to CO_2_ FAL and pulsed dye laser, and the other to pulsed dye laser with topical MEBO postoperatively.^65^^,^^84^ They both found that VSS scores significantly improved in the LADD than comparator groups (see Table 1). Lei et al. conducted a single-group trial investigating a new FAL technique, which included MEBO LADD.^73^ This study found there were statistically significant improvements in scar outcomes from the beginning to the end of treatment. Ge et al.’s study included any patients with scars ≥20% of TBSA.^33^ Some scars were hypertrophic, but their outcomes were not reported separately.

### Other compounds

Other LADD compounds identified were 5-aminolevulenic acid, asiaticoside, allantoin, botulinum toxin type A, DA-5520 (5% allantoin, 500 IU heparin sodium and 100 mg dexpanthenol), platelet-rich plasma, liquid silicone gel and verapamil.^40^^,^^41^^,^^52^^,^^56^^,^^58^^,^^60^^,^^71^^,^^72^ Each of these compounds had one publication devoted to it, and most of these aimed to investigate the efficacy of the laser-assisted delivery of their compound to another scar management modality (see Table 1).

Studies most frequently used the VSS to assess outcomes, with 4 publications suggesting some superiority of LADD treatment over comparator groups (see Table 1). The abstract, describing liquid silicone gel LADD, provided an unclear description of results, indicating that liquid silicone gel LADD was statistically superior in outcomes to laser or topical liquid silicone gel alone, but then stated that “there was no statistically significant difference between all treatments.”^41^

### Combination treatments

Four publications described using multiple LADD compounds at once to treat hypertrophic scars (see Table 1). One was an RCT, where authors compared the effect of different energy and density settings of CO_2_ FAL on clinical outcomes.^68^ A combination of heparin and allantoin gel was applied topically after each FAL treatment, and thereafter 3 times a day. All groups had a statistically significant improvement of their VSS scores compared to pretreatment.

The other 3 publications (a case report, a case vignette in a book chapter and a case series abstract) all used 5-FU and corticosteroid LADD (triamcinolone in 2 publications, unspecified steroid in one). The case report provided some descriptive results, but the abstract and book chapter did not.^46^^,^^53^^,^^59^

## DISCUSSION

This review identified 55 publications discussing LADD for the treatment of hypertrophic scars, using 16 different LADD substances (and various combinations of them). These compounds were delivered using 3 types of FALs (CO_2_, Er:YAG and erbium-doped yttrium-scandium-garnet-gallium). Study designs and comparison groups varied significantly, which, combined with the variety of LADD agents and lasers, and in some cases poor methodology reporting, made direct comparison of results difficult. Overall, there is insufficient high-quality evidence to suggest that LADD produced better patient outcomes than comparison treatments, and no consistent indications which scar characteristics it improves. Further large-scale trials with validated patient outcome measures are necessary to gain a better understanding of the role of this technique in hypertrophic scar management.

### Outcome measures for LADD efficacy

Studies used various outcome measures when assessing the efficacy of LADD in treating hypertrophic scars. The VSS and POSAS were the most common validated scar evaluation measurements, used in 29 studies. Both these scales are multifaceted (with POSAS incorporating patient perspectives) and provide a standardized method of scar assessment that can enable comparison of results between studies.^87–93^ However, the time-points at which these were used relative to the laser procedure varied between studies, and in some cases, the assessment scales were not used as designed. The rest of the publications used either single-faceted measures, such as caliper measurement of scar size, or unvalidated, subjective measures, such as a physician rating of scar photographs.^43^^,^^81^^,^^83^ This heterogenous outcome reporting complicates interpretation of results and meta-analysis of studies in future.^94^ Clinical research in this field would benefit from the use of validated scar assessment measurements for methodological robustness, replicability and comparison of study outcomes.

### Efficacy of LADD for treating hypertrophic scars

It is difficult to definitively comment on the efficacy of LADD for treating hypertrophic scars, not only due to the factors outlined above, but also because many studies have no comparison groups. The studies that compare LADD to other treatments demonstrate an improvement in various scar parameters in their LADD group, but do not unanimously demonstrate the superiority of any specific LADD substance. The results of the single-group studies are difficult to interpret, as LADD consists of 2 treatment components: the laser itself and the drug delivered. Without a comparison group of laser or drug alone, it is not possible to determine whether the effect on the scars treated with LADD is greater than the effect of laser by itself. We found 3 randomized studies of different LADD substances comparing LADD to FAL alone. These studies found that LADD produced superior outcomes.^52^^,^^56^^,^^65^ However, these are modest-sized studies, and investigation with larger cohorts would be prudent. Larger prospective trials comparing LADD to other treatment methods are needed, incorporating detailed methodology reporting and validated outcome measurement strategies.

A further challenge in assessing the efficacy of LADD for hypertrophic scar treatment is the variability in FAL and LADD procedural practices. It was noted that studies included in this review frequently omitted information regarding postoperative care practices, the dose of the LADD compound used, and the laser settings used. Although guidelines (such as the Template for Intervention Description and Replication [TIDieR] checklist) aim to improve the reporting of interventions in clinical trials, these are not always used.^95^^,^^96^ Furthermore, the TIDieR checklist does not necessarily capture periprocedural care variations, which are known to be common in clinical practice.^97^ Variations in these practices could potentially influence patient outcomes after LADD treatment, so are important to report and account for when interpreting and comparing trial results.

### Review strengths and limitations

This review identified a wide range of literature on the subject of LADD treatment of hypertrophic scars. By systematically reviewing study designs and outcomes, gaps in existing knowledge were highlighted and suggestions made regarding future research directions in this field.

By keeping the search criteria intentionally broad, many relevant studies were identified where LADD had not been used in the publication title, abstract or key words. This suggests that LADD is becoming almost a routine part of FAL scar treatment in some centers, and by some is not considered a potential confounding factor in interpreting outcomes of FAL treatment. Therefore, when interpreting the results of studies investigating FAL scar treatment, it may be of use to review study methodology to ascertain if LADD was used as part of the standard laser protocol.

This review had 4 main limitations. Firstly, studies that used emollients, silicone gel pads, or topical anti-infective agents were excluded. Whilst anti-infective agents and emollients are not typically used to treat hypertrophic scars, and the action of silicone gel pads has been shown not to be due to the instillation of silicone into tissues, there are no studies to show if these compounds exert a clinically significant effect on scars when used immediately after FAL.^98^ Secondly, only human studies were included, hence omitting LADD compounds that had not reached human trials. Preclinical studies could be a focus of a review in future. Thirdly, this review elected to combine the terms “hypertrophic scar” with “keloid,” which could superimpose a confounder on the results, given that although these entities are difficult to clinically distinguish, they have several differences in their biology.^99^ Individual review of LADD for hypertrophic scars and keloids could be pursued in future. Finally, it is it is important to acknowledge that the measurement of scar outcomes is imperfect, and that “a consensus about the ideal scar scale or tool is still lacking,” as per a literature review on objective scar assessment by Brusselaers et al.^90^^,^^100^^,^^101^ Multifaceted scales, such as VSS and POSAS, are subject to intra- and inter-observer variability due to being based upon subjective assessment, focus on a limited area of the scar, and do not capture the full breadth of patient experience.^90^^,^^92^^,^^101^ Objective single-faceted measures likewise have their limitations, as they present a single aspect of the scar.^90^ Some objective tools, such as calipers, can also incompletely capture the parameter they seek to measure.^90^ For example, scar thickness extends both above and below the surface of the surrounding skin, hence calipers would not be able to completely capture this parameter, whereas high-frequency ultrasound could.^90^

## CONCLUSION

This scoping review has found that many substances are used for LADD treatment of hypertrophic scars, with a range of study methodologies and outcome measures used to assess the clinical efficacy of this treatment. Unfortunately, the heterogeneity of study methods and outcome reporting limits the replication of existing trials, the comparison of study outcomes, and the clinical translation of existing research. At present, the benefit of LADD to patients with hypertrophic scars needs to be investigated with further high-quality, well-reported, large studies to inform best practices.

## Supplementary Material

Supplementary_File_1_iraf167

Supplementary_File_2_iraf167

Supplementary_Table_1_iraf167

Supplementary_Table_2_iraf167

## Notes

Author contributions: Maria Shilova (Conceptualization [equal], Data curation, Formal analysis, Investigation [lead], Methodology [equal], Project administration, Writing—original draft, Writing—review & editing [lead]), Karin Plummer (Conceptualization [supporting], Supervision, Writing—review & editing [equal]), Robert Ware (Conceptualization [supporting], Supervision, Writing—review & editing [equal]), Roy Kimble (Conceptualization, Supervision [equal], Writing—review & editing [supporting]), Justin Clark (Methodology, Resources, Writing—review & editing [supporting]), Esther Cho (Investigation [equal], Writing—review & editing [supporting]), Lucinda McMillan (Investigation [equal], Writing—review & editing [supporting]), Laura Kimble (Investigation [equal], Writing—review & editing [supporting]), Brandon Meikle (Investigation [equal], Writing—review & editing [supporting]), Lauren Kunde (Writing—review & editing [supporting]), and Bronwyn Griffin (Conceptualization, Investigation, Methodology [equal], Supervision [lead], Validation, Writing—review & editing [equal])

Funding: None declared.
