# Supplementary material for: Laser-Assisted Drug Delivery for Hypertrophic Scar Treatment: A Scoping Review
Source: J Burn Care Res. 2025 Sep 13;47(1):130–46. doi: 10.1093/jbcr/iraf167 (PMC12770983; doi:10.1093/jbcr/iraf167)
Supplement: Supplementary_File_1_iraf167 [file supplementary_file_1_iraf167.docx]

## Supplementary File 1: literature search strategies

PubMed

("Cicatrix, Hypertrophic"[Mesh] OR "Hypertrophic cicatrices"[tiab] OR "Hypertrophic cicatrix"[tiab] OR "Hypertrophic Scar"[tiab] OR "Hypertrophic scars"[tiab] OR HTS[tiab])

AND

("Lasers"[Mesh] OR "Laser Therapy"[Mesh] OR Laser[tiab] OR Lasers[tiab])

AND

(“randomized controlled trial”[pt] OR “controlled clinical trial”[pt] OR randomized[tiab] OR randomised[tiab] OR placebo[tiab] OR randomly[tiab] OR trial[tiab] OR groups[tiab] OR Crossover[tiab] OR "Comparative Study"[pt] OR "Evaluation Study"[pt] OR "Case reports"[pt] OR "Epidemiologic Studies"[Mesh] OR “case-control studies”[Mesh] OR “Cohort Studies”[Mesh] OR “case control”[tiab] OR "Case study"[tiab] OR "Case series"[tiab] OR Cohort[tiab] OR “Follow up”[tiab] OR Observational[tiab] OR Longitudinal[tiab] OR Prospective[tiab] OR retrospective[tiab] OR “cross sectional”[tiab] OR “Cross-Sectional Studies”[Mesh] OR Investigated[tiab] OR Evaluated[tiab] OR Impact[tiab] OR Analysis[tiab] OR Statistics[tiab] OR Data[tiab] OR "statistics and numerical data"[sh] OR "epidemiology"[sh])

Cochrane CENTRAL

([mh "Cicatrix, Hypertrophic"] OR "Hypertrophic cicatrices":ti,ab OR "Hypertrophic cicatrix":ti,ab OR "Hypertrophic Scar":ti,ab OR "Hypertrophic scars":ti,ab OR HTS:ti,ab)

AND

([mh Lasers] OR [mh "Laser Therapy"] OR Laser:ti,ab OR Lasers:ti,ab)

Embase

("hypertrophic scar"/exp OR "Hypertrophic cicatrices":ti,ab OR "Hypertrophic cicatrix":ti,ab OR "Hypertrophic Scar":ti,ab OR "Hypertrophic scars":ti,ab OR HTS:ti,ab)

AND

(laser/exp OR "laser therapy"/exp OR Laser:ti,ab OR Lasers:ti,ab)

AND

(random* OR factorial OR crossover OR placebo OR blind OR blinded OR assign OR assigned OR allocate OR allocated OR 'crossover procedure'/exp OR 'double-blind procedure'/exp OR 'randomized controlled trial'/exp OR 'single-blind procedure'/exp OR 'epidemiology'/exp OR 'controlled study'/exp OR 'cohort analysis'/exp OR "case control":ti,ab OR Cohort:ti,ab OR "Follow up":ti,ab OR Observational:ti,ab OR longitudinal:ti,ab OR Prospective:ti,ab OR retrospective:ti,ab OR "cross sectional":ti,ab OR 'Cross-Sectional Studies'/exp OR Investigated:ti,ab OR Analysis:ti,ab OR Statistics:ti,ab OR Data:ti,ab OR "Case study":ti,ab OR "Case series":ti,ab)

WHO International Clinical Trials Registry Platform (ICTRP) and Clinicaltrials.gov searched via Cochrane (see Cochrane CENTRAL search strategy above)
