# Supplementary material for: Laser-Assisted Drug Delivery for Hypertrophic Scar Treatment: A Scoping Review
Source: J Burn Care Res. 2025 Sep 13;47(1):130–46. doi: 10.1093/jbcr/iraf167 (PMC12770983; doi:10.1093/jbcr/iraf167)
Supplement: Supplementary_File_2_iraf167 [file supplementary_file_2_iraf167.docx]

## Supplementary File 2 A full PRISMA flow diagram detailing the original and updated literature searches

Records identified from database search (n = 909)

*PubMed, Embase, Cochrane databases*

Duplicate records removed (n = 340)

Records screened by two authors (n = 569)

Records excluded

(n = 539)

Included records (n = 30)

Backwards and forwards reference searching

(n = 626)

Duplicate records removed (n = 46)

Records screened by two authors (n = 580)

Included articles from reference searching (n = 19)

Total studies included in review from first search

(n = 49)

Records identified from database search (n = 188)

*PubMed, Embase, Cochrane databases*

Records screened by two authors (n = 131)

Included records (n = 6)

Backwards and forwards reference searching (n = 104)

Records screened by two authors (n = 104)

Included articles from reference searching (n = 0)

Total studies included in review from updated search (n = 6)

Duplicate records removed (n = 57)

Records excluded

(n = 125)

Duplicate records removed (n = 0)

Total studies included in review (n = 55)

Identification

Screening

Identification

Screening

Included

*Search December 2021*

*Search February 2023*

Records excluded

(n = 561)

Records excluded

(n = 104)
