# Supplementary material for: Laser-Assisted Drug Delivery for Hypertrophic Scar Treatment: A Scoping Review
Source: J Burn Care Res. 2025 Sep 13;47(1):130–46. doi: 10.1093/jbcr/iraf167 (PMC12770983; doi:10.1093/jbcr/iraf167)
Supplement: Supplementary_Table_1_iraf167 [file supplementary_table_1_iraf167.docx]

## Supplementary Table 1: data charting items

| **Data category** | **Specific items** |
| --- | --- |
| Publication details | First author |
|  | Year of publication |
|  | Origin/country where the study was conducted |
|  | Type of publication |
| Publication aims | - |
| Study design |  |
| Population | Type of population (i.e. adult, paediatric, mixed) |
|  | Total sample size |
|  | Mean age |
|  | % female, % male |
|  | Number of groups |
|  | Group size |
|  | Mean age of groups |
|  | % female, % male in groups |
| Scars | Scar origin |
|  | Average timing of laser post-injury |
| Laser | Type of laser |
|  | Make/model |
|  | Laser settings |
| LADD substance | Name of medication/substance |
|  | Concentration |
|  | Dose |
|  | Timing of application |
|  | Method of application (e.g. topical with dressing, topical without dressing) |
| Study outcomes | Method of measuring outcomes |
|  | Adverse events |
