# Supplementary material for: Laser-Assisted Drug Delivery for Hypertrophic Scar Treatment: A Scoping Review
Source: J Burn Care Res. 2025 Sep 13;47(1):130–46. doi: 10.1093/jbcr/iraf167 (PMC12770983; doi:10.1093/jbcr/iraf167)
Supplement: Supplementary_Table_2_iraf167 [file supplementary_table_2_iraf167.docx]

## Supplementary Table 2. Mixed Methods Appraisal Tool quality assessment of full-text studies investigating the efficacy of laser-assisted drug delivery for the treatment of hypertrophic scars

|  | General | | Qualitative | | | | | Quantitative RCTs | | | | | Quantitative, non-randomised | | | | | Qualitative, descriptive | | | | |  |
| --- | --- | --- | --- | --- | --- | --- | --- | --- | --- | --- | --- | --- | --- | --- | --- | --- | --- | --- | --- | --- | --- | --- | --- |
| _Article_ ^Question^ | S1 | S2 | 1.1 | 1.2 | 1.3 | 1.4 | 1.5 | 2.1 | 2.2 | 2.3 | 2.4 | 2.5 | 3.1 | 3.2 | 3.3 | 3.4 | 3.5 | 4.1 | 4.2 | 4.3 | 4.4 | 4.5 | Total |
| Abd El-Dayem^50^ | 1 | 0 | - | - | - | - | - | - | - | - | - | - | 1 | 0 | C | C | C | - | - | - | - | - | 1 |
| Al Janahi^a 75^ | 1 | 1 | - | - | - | - | - | - | - | - | - | - | - | - | - | - | - | 1 | 1 | 0 | 1 | - | 3 |
| Behrangi^55^ | 1 | 1 | - | - | - | - | - | - | - | - | - | - | 1 | 0 | C | C | C | - | - | - | - | - | 1 |
| Burns^37^ | 1 | 0 | 1 | C | 0 | 1 | 1 | - | - | - | - | - | - | - | - | - | - | - | - | - | - | - | 3 |
| Cavalié^43^ | 1 | 1 | - | - | - | - | - | - | - | - | - | - | - | - | - | - | - | 1 | 1 | 0 | 1 | 0 | 3 |
| Dai^52^ | 1 | 1 | - | - | - | - | - | - | - | - | - | - | 1 | 0 | C | 1 | C | - | - | - | - | - | 2 |
| Elrod^54^ | 1 | 1 | - | - | - | - | - | - | - | - | - | - | 1 | 1 | 1 | 1 | 0 | - | - | - | - | - | 4 |
| Ge^33^ | 1 | 0 | - | - | - | - | - | - | - | - | - | - | 1 | 0 | 1 | 0 | 0 | - | - | - | - | - | 2 |
| Han^58^ | 1 | 1 | - | - | - | - | - | C | 1 | 1 | 1 | C | - | - | - | - | - | - | - | - | - | - | 3 |
| Khandelwal^67^ | 1 | 1 | - | - | - | - | - | - | - | - | - | - | 0 | 1 | C | 0 | 0 | - | - | - | - | - | 1 |
| Krakowski^a 44^ | 1 | 1 | - | - | - | - | - | - | - | - | - | - | - | - | - | - | - | 1 | C | C | 1 | - | 2 |
| Lei^73^ | 1 | 1 | - | - | - | - | - | - | - | - | - | - | C | 0 | C | 0 | 0 | - | - | - | - | - | 0 |
| Lin^48^ | 1 | 1 | - | - | - | - | - | C | 1 | 1 | 0 | 1 | - | *-* | *-* | *-* | *-* | - | - | - | - | - | 3 |
| Lin^61^ | 1 | 1 | - | - | - | - | - | - | - | - | - | - | 0 | 1 | 1 | 0 | 1 | - | - | - | - | - | 3 |
| Liu^65^ | 1 | 0 | - | - | - | - | - | C | C | C | C | C | - | - | - | - | - | - | - | - | - | - | 0 |
| Liu^82^ | 1 | 1 | - | - | - | - | - | - | - | - | - | - | C | 1 | C | 1 | 0 | - | - | - | - | - | 2 |
| Lv^13^ | 1 | 1 | - | - | - | - | - | - | - | - | - | - | 0 | 1 | 1 | 0 | C | - | - | - | - | - | 2 |
| Majid^80^ | 1 | 0 | - | - | - | - | - | - | - | - | - | - | C | 0 | 1 | 0 | C | - | - | - | - | - | 1 |
| Maninder^35^ | 1 | 0 | - | - | - | - | - | - | - | - | - | - | C | 0 | 1 | 0 | 0 | - | - | - | - | - | 1 |
| Manuskiatti^57^ | 1 | 1 | - | - | - | - | - | 1 | 1 | 1 | C | C | - | - | - | - | - | - | - | - | - | - | 3 |
| Ouyang^84^ | 1 | 1 | - | - | - | - | - | C | C | 1 | C | C | - | - | - | - | - | - | - | - | - | - | 1 |
| Park^36^ | 1 | 0 | - | - | - | - | - | - | - | - | - | - | 0 | 0 | 1 | 1 | C | - | - | - | - | - | 2 |
| Sabry^60^ | 1 | 0 | - | - | - | - | - | C | 1 | C | C | C | - | - | - | - | - | - | - | - | - | - | 1 |
| Sabry^56^ | 1 | 0 | - | - | - | - | - | C | 1 | 1 | C | C | - | - | - | - | - | - | - | - | - | - | 2 |
| Saraiva^39^ | 1 | 1 | - | - | - | - | - | - | - | - | - | - | 0 | 0 | 1 | 0 | C | - | - | - | - | - | 1 |
| Tan^76^ | 1 | 1 | - | - | - | - | - | - | - | - | - | - | C | 1 | 1 | 0 | C | - | - | - | - | - | 2 |
| Tawfik^78^ | 1 | 0 | - | - | - | - | - | C | 1 | 1 | C | C | - | - | - | - | - | - | - | - | - | - | 2 |
| Tu^a 38^ | 1 | 1 | - | - | - | - | - | - | - | - | - | - | - | - | - | - | - | C | 1 | C | 0 | - | 1 |
| Waibel^83^ | 1 | 0 | - | - | - | - | - | - | - | - | - | - | 0 | 0 | 1 | 1 | C | - | - | - | - | - | 2 |
| Waibel^81^ | 1 | 0 | - | - | - | - | - | - | - | - | - | - | C | 0 | C | 1 | C | - | - | - | - | - | 1 |
| Wang^66^ | 1 | 1 | - | - | - | - | - | - | - | - | - | - | 1 | 1 | 1 | 0 | 1 | - | - | - | - | - | 4 |
| Yan^40^ | 1 | 0 | - | - | - | - | - | C | 0 | C | 1 | C | - | - | - | - | - | - | - | - | - | - | 1 |
| Ządkowski^71^ | C | C | - | - | - | - | - | - | - | - | - | - | C | 0 | C | 0 | 0 | - | - | - | - | - | 0 |
| Zhang^68^ | 1 | 1 | - | - | - | - | - | C | C | C | C | C | - | - | - | - | - | - | - | - | - | - | 0 |
| Scores of 1 were allocated to “yes” responses, 0 for “no” responses, and C for “can’t tell” responses, which was valued at 0.  a. case study/series, hence question 4.5 (regarding appropriateness of statistical analysis techniques undertaken) is not applicable | | | | | | | | | | | | | | | | | | | | | | | |
